# Autophagy dysfunction contributes to NLRP1 inflammasome-linked depressive-like behaviors in mice

**DOI:** 10.1186/s12974-023-02995-4

**Published:** 2024-01-04

**Authors:** Ya-Jing Zhu, Jing Huang, Ru Chen, Yu Zhang, Xin He, Wen-Xin Duan, Yuan-Lei Zou, Meng-Mei Sun, Hui-Li Sun, Si-Min Cheng, Hao-Chuan Wang, Hao Zhang, Wen-Ning Wu

**Affiliations:** 1 Department of Pharmacology & Research Centre for Neurological Disorders, School of Basic Medical Sciences, Anhui Medical University, Hefei, 230032 People’s Republic of China; 2 Department of Oncology, the First Affiliated Hospital of Anhui Medical University, Hefei, 230032 People’s Republic of China; 3 Key Laboratory of Anti-Inflammatory and Immunopharmacology, Anhui Medical University, Hefei, 230032 People’s Republic of China

**Keywords:** MDD, NLRP1 inflammasome, Autophagy, CSDS, mTOR, PI3K/AKT

## Abstract

**Background:**

Major depressive disorder (MDD) is a common but severe psychiatric illness characterized by depressive mood and diminished interest. Both nucleotide-binding oligomerization domain, leucine-rich repeat and pyrin domain-containing 1 (NLRP1) inflammasome and autophagy have been reported to implicate in the pathological processes of depression. However, the mechanistic interplay between NLRP1 inflammasome, autophagy, and depression is still poorly known.

**Methods:**

Animal model of depression was established by chronic social defeat stress (CSDS). Depressive-like behaviors were determined by social interaction test (SIT), sucrose preference test (SPT), open field test (OFT), forced swim test (FST), and tail-suspension test (TST). The protein expression levels of NLRP1 inflammasome complexes, pro-inflammatory cytokines, phosphorylated-phosphatidylinositol 3-kinase (p-PI3K)/PI3K, phosphorylated-AKT (p-AKT)/AKT, phosphorylated-mechanistic target of rapamycin (p-mTOR)/mTOR, brain-derived neurotrophic factor (BDNF), phosphorylated-tyrosine kinase receptor B (p-TrkB)/TrkB, Bcl-2-associated X protein (Bax)/B-cell lymphoma-2 (Bcl2) and cleaved cysteinyl aspartate-specific proteinase-3 (caspase-3) were examined by western blotting. The mRNA expression levels of pro-inflammatory cytokines were tested by quantitative real-time PCR. The interaction between proteins was detected by immunofluorescence and coimmunoprecipitation. Neuronal injury was assessed by Nissl staining. The autophagosomes were visualized by transmission electron microscopy. *Nlrp1a* knockdown was performed using an adeno-associated virus (AAV) vector containing *Nlrp1a*–shRNA–eGFP infusion.

**Results:**

CSDS exposure caused a bidirectional change in hippocampal autophagy function, which was activated in the initial period but impaired at the later stage. In addition, CSDS exposure increased the expression levels of hippocampal NLRP1 inflammasome complexes, pro-inflammatory cytokines, p-PI3K, p-AKT and p-mTOR in a time-dependent manner. Interestingly, NLRP1 is immunoprecipitated with mTOR but not PI3K/AKT and CSDS exposure facilitated the immunoprecipitation between them. Hippocampal *Nlrp1a* knockdown inhibited the activity of PI3K/AKT/mTOR signaling, rescued the impaired autophagy and ameliorated depressive-like behavior induced by CSDS. In addition, rapamycin, an autophagy inducer, abolished NLRP1 inflammasome-driven inflammatory reactions, alleviated depressive-like behavior and exerted a neuroprotective effect.

**Conclusions:**

Autophagy dysfunction contributes to NLRP1 inflammasome-linked depressive-like behavior in mice and the regulation of autophagy could be a valuable therapeutic strategy for the management of depression.

**Supplementary Information:**

The online version contains supplementary material available at 10.1186/s12974-023-02995-4.

## Background

Major depressive disorder (MDD) is a severe psychiatric illness characterized by depressive mood and diminished interest or anhedonia, and it is often accompanied by impaired cognitive function and vegetative symptoms [1, 2]. This disorder affects more than 300 million people and has been considered as the leading cause of disability and disease burden worldwide [3]. Although a variety of antidepressants are available for MDD treatment, less than half of the depressed patients achieve complete remission upon therapy [4]. Therefore, a deeper understanding of the pathogenic events and molecular changes such as inflammation and autophagy that occur during depression is needed to develop better therapeutic strategies for these patients.

Accumulating evidence from clinical and experimental studies indicates that inflammation and depression are closely interrelated [5, 6]. MDD patients and experimental animals exhibit increased inflammatory processes, whereas there is an improvement in depressive symptoms with anti-inflammatory treatments [7–9]. Inflammation has been considered as a critical component of depressive pathophysiology, which is involved in the onset and maintenance of MDD [10]. Inflammasomes, a crucial component of the immune inflammatory process, are multiprotein complexes that consist of an upstream sensor NOD-like receptor (NLR) family, the adaptor apoptosis-associated speck-like protein containing a caspase-activating recruitment domain (ASC), and the downstream effector caspase-1. The activity of inflammasomes can trigger a series of inflammatory reactions and link to various central nervous system (CNS) disorders [11, 12]. NLRP1, the first characterized inflammasome, is mainly presented in neurons and has been reported to implicate in many neurological disorders, such as brain injury, neurodegenerative diseases, nociception, and epilepsy [13, 14]. Our previous study showed that NLRP1 inflammasome is involved in chronic stress-induced depressive-like behaviors in mice [15]. However, the precise mechanism of how NLRP1 inflammasome participates in the development of depression needs to be further clarified.

Autophagy is a highly conserved and regulated catabolic process that degrades damaged proteins and organelles through autophagosome formation. It is essential for maintaining homeostasis and the function of the CNS [16]. Moderate autophagy is considered as a protective mechanism during the development of neurological disorders, while insufficient or excessive autophagy can be harmful to neurons [17–19]. Autophagic dysfunction has been implicated in several neurological disorders, including depression [20, 21]. Mounting evidence reveals the inter-regulation of inflammasomes and autophagy. Autophagy can negatively regulate inflammasome activation in an attempt to protect the host from excessive inflammation [22]. Similarly, autophagy is restricted or diminished in inflammatory diseases accompanied by excessive inflammation and hyperactivation of inflammasomes [23–25]. Recently, the mutual regulation of NLRP1 inflammasome and autophagy has been reported in oxidative stress damage and myocardial infarction [26, 27]. However, their interaction and the underlying molecular mechanism in depression remain unclear. Consequently, we investigated the interplay of NLRP1 inflammasome and autophagy in an animal model of depression induced by chronic social defeat stress (CSDS). Our results show that autophagy is activated in the early stage of CSDS and is impaired at the later stage, resulting from the activity of NLRP1–PI3K/AKT/mTOR pathway, which contributes to NLRP1 inflammasome-linked depressive-like behaviors in mice.

## Materials and methods

### Animals

Adult C57BL/6J male mice (7–8 weeks, 20–23 g) and single-housed male CD1 mice (13–15 weeks, sexually experienced retired breeders) were purchased from the Experimental Animal Center of Anhui Medical University. Before the experiments, the animals were housed under controlled conditions at an appropriate temperature (22 ± 2 °C), humidity (60%), and a 12-h light/12-h dark cycle (lights-on from 8:00 to 20:00). Food and water were available ad libitum. All animal experiments were conducted during light phase and performed according to the protocols approved by the Committee for Experimental Animal Use and Care of Anhui Medical University.

### Chemicals

Primary antibodies against NLRP1 (ab3683), cysteinyl aspartate-specific proteinase-1 (caspase-1) (ab1872), Beclin-1 (ab62557), microtubule-associated proteins light chain 3 (LC3) (ab48394) and BDNF (ab108319) were purchased from Abcam (San Francisco, CA, USA). autophagy-related protein 5 (Atg5) (#12994), autophagy-related protein 7 (Atg7) (#2631), prostacyclin (p62) (#5114), Interleukin-6 (IL-6) (#12912), Interleukin-1β (IL-1β) (#12703), tumor necrosis factor-α (TNF-α) (#3707), cleaved caspase-3 (#9664), p-PI3K (#4228), PI3K (#4249), p-AKT (#4060), AKT (#9272), p-mTOR (#2971), mTOR (#2983), p-TrkB (#4619), TrkB (#4603), Bcl-2 (#3498) and Bax (#2772) were purchased from Cell Signaling Technology (CST, Beverly, MA, USA), while an antibody against apoptosis-associated speck-like protein containing a caspase-activating recruitment domain (ASC) (sc-33958) was purchased from Santa Cruz Biotechnology (Santa Cruz, CA, USA). Horseradish peroxidase-conjugated secondary antibodies were purchased from Santa Cruz Biotechnology (Santa Cruz, CA, USA). Other general agents were commercially available.

### Chronic stress procedures

Chronic social defeat stress (CSDS) was performed as previously reported [28, 29]. There were three groups of C57BL6/J mice (9 in each group) and they were exposed to CSDS for different durations of 3 days, 7 days, and 10 days. Before CSDS, all CD1 mice were screened for aggressiveness before use according to published protocols. Three groups of C57BL6/J mice were then transferred to the home cage of a novel CD1 mouse for 5–10 min daily each day. After the social defeat session, C57BL6/J mice were housed in one half of the cage and CD1 mice in the other half of the cage were separated by a perforated Plexiglas barrier to allow continuous psychological stress from sensory cues for the next 24 h. After the completion of the protocol, the defeated mice were kept alone overnight, and the social interaction test was performed at the following day.

### Rapamycin treatment

Rapamycin powder (MCE, USA, HY-10219) was dissolved in ethanol and stored at a stock concentration of 25 mg/ml in aliquots at − 20 °C. Prior to use, the working solution was freshly prepared by dissolving rapamycin in a mixture of 4% ethanol, 5% Tween 80, and sterile saline. C57BL6/J mice (9 in each group) received intraperitoneal injection of rapamycin (2 mg/kg) at 30 min prior to CSDS once daily for 10 days.

### Open field test (OFT)

The open field test was performed under dim light to study the differences in general motor activity between mice. Mice were placed in a floor open field with clear sidewalls (96 × 96 × 50 cm) and left free to explore the field for 5 min. First, the mouse was placed in the center area and allowed to adapt for 2 min. Then, the locomotor activity was recorded for 3 min. The field was thoroughly cleaned with 75% ethanol between trials.

### Social interaction test (SIT)

The social interaction test was performed as described previously [29]. Mice were placed in a white plastic open box (area size 45 × 45 × 45 cm) with a transparent perforated plastic compartment on one side of the open arena (area size 8 × 6 cm). Many small holes in the plastic compartment allow defeated mice to smell the unfamiliar CD1 mice. Sessions were videotaped, recording the time the mice spent in each social contact area, defined as a 24 × 14 cm square area around each compartment. Mice were placed over two 150 s trials. During the first detection, the perforated plastic compartment was empty. In the second detection, an unfamiliar CD1 was placed in it. The social interaction ratio = time spent in an interaction zone with a CD1 mouse/time spent in an interaction zone without a CD1 mouse. Mice with a social interaction ratio less than 1 were defined as “susceptible”, and mice with a social interaction ratio greater than 1 were considered “resilient”.

### Sucrose preference test (SPT)

The sucrose preference test (SPT) was performed to observe the anhedonic response in mice. Twelve hours before testing, all mice were deprived of water and food. Mice were single-caged during the test. Two standard drinking bottles containing regular water or 1% sucrose water were provided in the cage simultaneously, and the mice had free access to water for the next 24 h. The bottle positions were switched every 6 h to avoid location preference. The sucrose preference rate was calculated as 1% sucrose water consumption/(total liquid consumption) × 100%.

### Tail suspension test (TST)

Mice were individually suspended by their tails to a magnet using medical tape and placed in the corresponding chamber of a specially manufactured tail suspension box with the mice heads down. Auditory and visual isolation was performed on the mice. After the mice adapted to the tail suspension for 2 min in the chamber, the tail suspension experimental instrument (Techman, TST-100, Chengdu, China) was used for monitoring [30]. Immobility time was recorded for 4 min. At the end of the experiment, the experimental data were derived from analyzing the differences in the immobility time of mice in each group.

### Forced swim test (FST)

The mice were placed in the water to provide an unavoidable pressure environment to detect depressive behavior. The mice were placed individually into a Plexiglas cylinder filled (25 cm in diameter) with water (maintained at 24 ± 1 °C) approximately 30 cm in depth. A video monitored the formal test. Each mouse was allowed to adapt for 2 min. The videos of the swim test were taken in the following 4 min section. The ANY-maze software was used to record the immobility time of the mice.

### Western blotting

Western blotting was performed using standard methods. In brief, after the last behavioral test, the animals were sacrificed by decapitation and the hippocampus was isolated from the brain on the ice. Dissected hippocampal tissues were homogenized in lysis buffer. The homogenate was centrifuged at 12,000 rpm at 4 °C for 15 min, and the supernatant was separated for Western blotting. The protein concentration was evaluated by a BCA protein assay kit (Pierce Biotechnology, Inc., Rockford, USA). Equal amounts of protein from each sample were separated by electrophoresis and then transferred onto PVDF membranes (Millipore) and blocked with 5% BSA dissolved in stripping solution at room temperature for 1 h. Subsequently, the membrane was rinsed and incubated overnight at 4 °C with different primary antibodies (anti-NLRP1, anti-Beclin-1, anti-LC3, anti-Atg5, anti-Atg7, anti-p62, anti-IL-1β, anti-IL-6, anti-TNF-α, anti-p-PI3K, anti-PI3K, anti-p-AKT, anti-p-AKT, anti-p-mTOR, anti-mTOR, anti-p-TrkB, anti-TrkB, anti-Bax and anti-Bcl2, 1:1000 dilution; anti-BDNF, 1:2000 dilution; anti-caspase-3 (cleaved), 1:800 dilution; anti-caspase-1, 1:500 dilution; anti-ASC, 1:200 dilution). After rinsing, the corresponding secondary antibody was added to incubate the membranes for 1 h at 37 °C. The membranes were visualized by enhanced chemiluminescence (Amersham Pharmacia Biotech, Inc., Piscataway, NJ, USA) on an imaging system (Bio-Rad, USA).

### Quantitative real-time PCR analysis

The animals were sacrificed by decapitation after the final behavioral tests, and total RNA was extracted from hippocampus using TRIzol reagent (Invitrogen, USA) as per the manufacturer’s instructions. Subsequently, cDNA was synthesized using the PrimeScript First Strand cDNA Synthesis Kit (Takara Biotechnology, Japan). PCR amplification of the cDNA was then carried out using standard procedures. Specific primers were employed for amplification, including IL-6 (forward: 5′-GAGAGGAGACTTCACAGAGGATACC-3′，reverse: 5′-TCATTTCCAC GATTTCCCAGAGAAC-3′), IL-1β (forward: 5′-CACTACAGGCTCCGAGATGAACAAC-3′, reverse: 5′-TGTCGTTGCTTGGTTCTCCTTGTAC-3′), TNF-a (forward: 5′-GCCTCTTCT CATTCCTGCTTGTGG-3′, reverse: 5′-GTGGTTTGTGAGTGTGAGGGTCTG-3′), β-actin (forward: 5′-TTCCTTCCTGGGTATGGAAT-3′, reverse: 5′-GAGG AGCAATGATCTTGAT C-3′). Each experimental group was performed in triplicate, using β-actin as the internal reference. Fluorescent signals were captured during the extension stage, and the Ct values of the samples were calculated. Data analysis was conducted using the 2^−ΔΔCT^ method.

### Injection of adenovirus-associated vector (AAV)

An adeno-associated viral vector (AAV) was used to knockdown mouse NLRP1 (*Nlrp1a*-shRNA). A second construct (control-shRNA) lacking the NLRP1 sequence served as a control (Hanbio, Shanghai, China). In brief, *Nlrp1a*-shRNA or control-shRNA was cloned into pHBAAV–U6–MCS–CMV–eGFP (AAV2/9, 1.0 × 1012 TU/ml) and confirmed by sequencing. Preparation and purification of the recombinant plasmids were performed by triple transfection, helper-free method, and purification. The sequences for scrambled control-shRNA and *Nlrp1a*-shRNA were 5′-TTCTCCGAACGTGTCACGTAA-3′ and 5′-CAGCTAGAGAGGAACTTG AAGCTAA-3′, respectively. Mice were randomly assigned to receive *Nlrp1a*-shRNA (10 mice) or control-shRNA (10 mice). Standard procedures for aseptic surgery were followed. Animals were anesthetized with isoflurane gas. The scalp was incised, the skin was retracted, and the skull was exposed. Holes were drilled through the skull above the hippocampus bilaterally (− 1.6 mm anteroposterior (AP), ± 1.8 mm mediolateral (ML), − 1.6 mm dorsoventral (DV)). For each injection, 1.5 μL of the vector was infused into the hippocampus at a rate of 0.15 μL/min using a 5-μL Hamilton syringe connected to a 30-gauge needle. The mice were kept on heating pads during the surgery and recovery procedures. After 4 weeks, six mice of *Nlrp1a*-shRNA and control-shRNA group received CSDS exposure. Other four mice were used to check the efficacy of transfection and silencing by immunofluorescence and western blotting.

### Transmission electron microscopy (TEM)

The autophagosomal structures in the hippocampal CA1 region were subjected to TEM analysis. Hippocampal tissue pieces of the CA1 region (from 3 mice in each group) were carefully isolated, rapidly cut out, diced into 1 mm^3^ cube, and then transferred to the 2.5% glutaral immediately. After fixation, the samples were dehydrated with graded ethanol solutions and embedded in Araldite. The ultrathin sections were stained with 4% uranyl acetate and examined by TEM (Leica EM UC7, Germany). Mice were randomly picked for observation.

### Immunofluorescence

Mice were deeply anaesthetized and transcardially perfused with 20 ml PBS followed by 20 ml of cold 4% paraformaldehyde (PFA). Brains (from 3 mice in each group) were then removed and post-fixed with the same 4% PFA solution for 4–6 h at 4 ℃. The samples were then transferred to 30% sucrose in PBS overnight. Sample Sects. (14 μm) were prepared on gelatin-coated glass slide via cryosection. The sections were then washed three times in PBS for 5 min each, blocked with 5% bovine serum albumin (BSA) and 0.2% Triton X-100 for 1 h at room temperature and then incubated with primary antibody against mTOR (CST, Beverly, MA, USA) or NLRP1 (Santa Cruz, CA, USA) at 4 °C overnight. After washing with phosphate-buffered solution (PBS), samples were incubated with appropriate secondary antibody at room temperature for 1 h and cell nuclei were stained with DAPI. Fluorescent images were captured with a Pannoramic MIDI scanner (3D HISTECH, Budapest, Hungary).

### Coimmunoprecipitation (Co-IP)

Briefly, mouse hippocampal tissue (from 3 mice in each group) was used for the coimmunoprecipitation assay. Tissue lysates were precleared with protein G or A beads. Then, the lysis buffer containing the complete protease inhibitors was incubated at 4 °C with the appropriate antibody overnight. Immunocomplexes were collected by binding to protein A/G Plus-Agarose beads (Santa Cruz Biotechnology), washed, and analyzed by immunoblotting.

### Nissl staining

5-μm paraffin coronal sections of the hippocampus (from 3 mice in each group) were washed with distilled water and stained with Nissl staining solution (Beyotime, China), incubated for 5 min at 37 °C. Then, sections were washed twice with distilled water for a few seconds, rinsed with 95% ethanol for 5 min and air dry. Sections were washed twice in xylene for 5 min each. After sealing with neutral balsam, the stained sections were examined under a Pannoramic MIDI scanner (3D HISTECH, Budapest, Hungary). Nissl-positive cells were calculated by Image-Pro Plus 6.0 software (Media Cybernetics, USA) by a person blinded to group assignment. Results are expressed as relative numbers of neurons.

### Statistical analysis

All data were analyzed with the statistical program SPSS 17.0 (Chicago, IL, USA). Data are expressed as means ± SEM. Differences between experimental and control groups were assessed using a one-way analysis of variance (ANOVA) with an additional Bonferroni post hoc test. *P* < 0.05 was considered statistically significant.

## Results

### CSDS exposure leads to autophagy dysfunction in mice

**Fig. 1:**
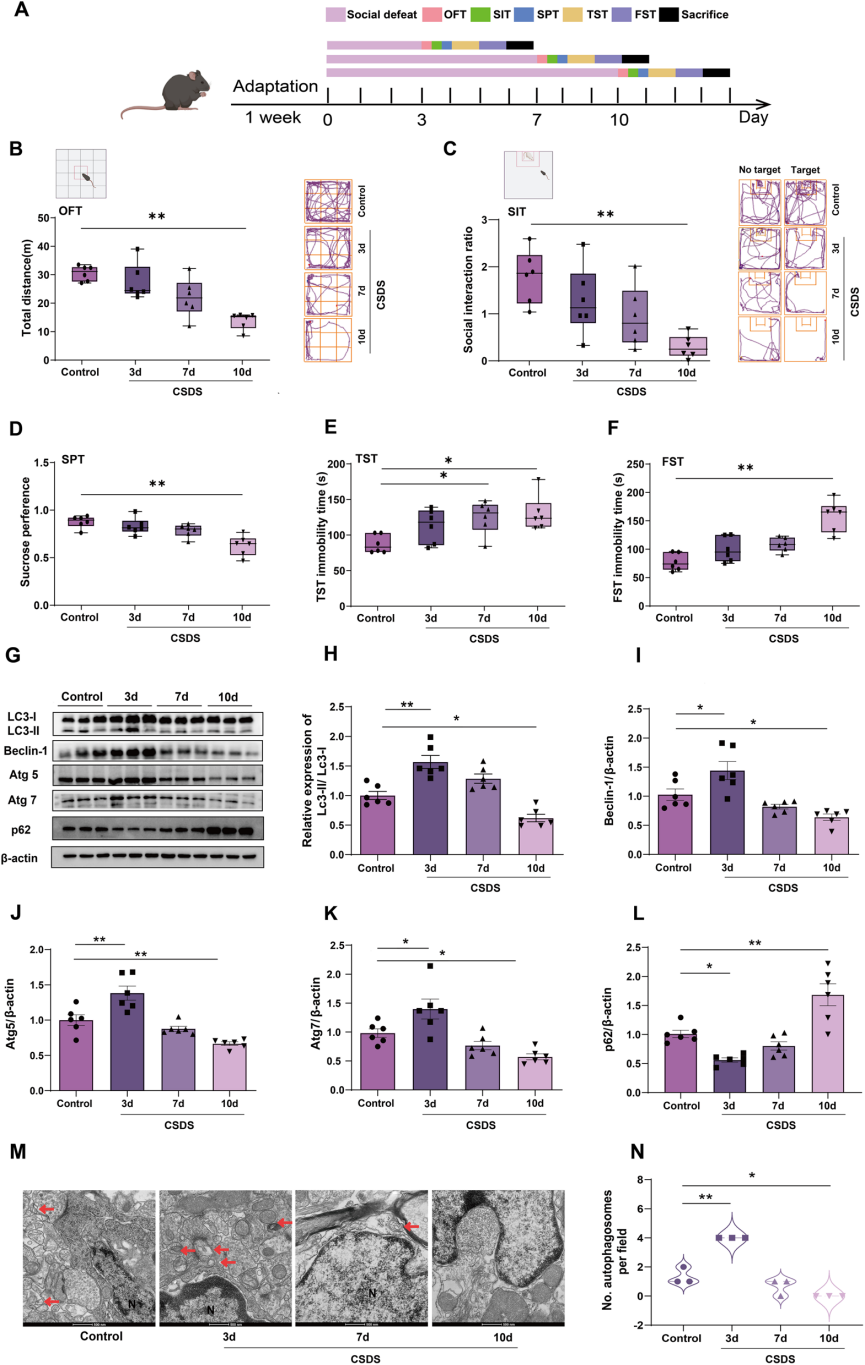
Effects of different time courses of CSDS exposure on autophagy function in mice. **A** Scheme of social defeat stress and behavior test. **B**, **C** Representative traces and statistical results show that CSDS exposure decreased the total moving distance (**B**) and the social interaction (**C**) in a time-dependent manner. **D** Statistical results show that CSDS decreased the sucrose performance in a time-dependent manner. **E**, **F** Statistical results show that CSDS exposure increased immobility time in TST (**E**) and FST (**F**) in a time-dependent manner. **G**–**L** Representative immunoreactive bands (**G**) and statistical results showing a bidirectional change in the expression of LC3-II/LC3-I (**H**), Beclin-1 (**I**), Atg5 (**J**), Atg7 (**K**), p62 (**L**) during CSDS. *n* = 6. (M–N) Representative images (**M**) and statistical results (**N**) showing a bidirectional change in the number of hippocampal autophagosomes (arrow) during CSDS. *n* = 3. Scale bar = 500 nm. Data were expressed as means ± SEM, *p < 0.05, **p < 0.01 vs. control group

Autophagy is pivotal in maintaining cell homeostasis in physiological as well as pathological situations [31], whereas neuropsychiatric conditions can cause significant impairments in cell homeostasis [32]. To determine the role of autophagy in depression, we first established an animal model using chronic social defeat stress (CSDS) (Fig. 1A). As shown in Fig. 1B–D (*n* = 6), the total distance, social interaction rate and sucrose preference were markedly decreased at 10 d after CSDS, while the immobility time in the TST and FST was significantly increased at the same timepoint (Fig. 1E, F, n = 6). These results suggest that CSDS caused significant depressive symptoms at 10 d in mice, which was not observed at 3 d or 7 d. Next, we investigated the effect of CSDS exposure on autophagy in mice by western blotting and transmission electron microscopy. Our results showed that the expression levels of hippocampal LC3-II/LC3-I, Beclin-1, Atg5 and Atg7 were increased at 3 d after CSDS exposure but decreased at 10 d, indicating that autophagy is activated at 3 d after CSDS exposure and impaired at 10 d in mice (Fig. 1G–K, n = 6). Consistently, a significant clearance of p62 was observed at 3 d after CSDS exposure, but a considerable accumulation was observed at 10 d (Fig. 1G, and L, n = 6). Similarly, the number of hippocampal autophagosomes was increased at 3 d after CSDS exposure but decreased at 10 d (Fig. 1M, N, *n* = 3). These results suggest that CSDS exposure causes a bidirectional change in autophagy function, which is activated in the initial period and impaired in the later period when depressive-like behavior was induced in mice.

### CSDS exposure activates NLRP1 inflammasome and PI3K/AKT/mTOR signaling pathway in mice

**Fig. 2:**
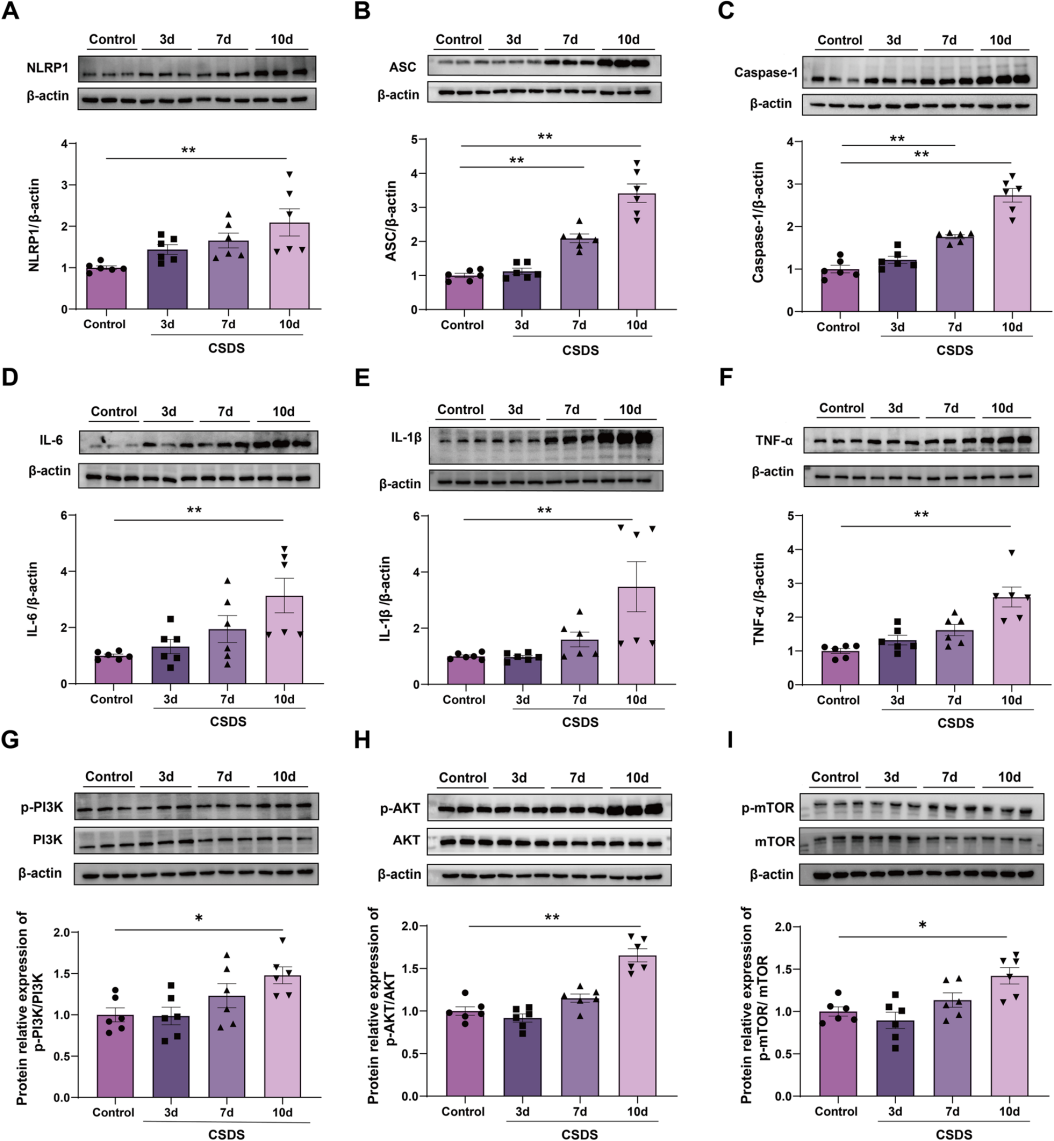
Effects of different time courses of CSDS on NLRP1 inflammasome and PI3K/AKT/mTOR signaling activation in mice. **A**–**C** Representative immunoreactive bands and statistical results show that CSDS exposure increased the expression of hippocampal NLRP1 (**A**), ASC (**B**), caspase-1 (**C**), IL-6 (**D**), IL-1β (**E**), TNF-α (**F**), p-PI3K (**G**), p-AKT (**H**) and p-mTOR (**I**) in a time-dependent manner. Data represented the mean ± SEM. *n* = 6, *p < 0.05, **p < 0.01 vs. control group

Recent evidence suggests strong links between autophagy, neuroinflammation, and depression [33]. To study the underlying mechanism of autophagy dysfunction under CSDS, we tested the expression of hippocampal NLRP1 inflammasome complexes in mice. Our results showed that CSDS exposure significantly increased the expression levels of hippocampal NLRP1, ASC and caspase-1 in a time-dependent manner, which was statistically significant at 10d after stress stimuli (Fig. 2A–C, *n* = 6). Moreover, the expression of proinflammatory cytokines such as IL-6, IL-1β, and TNF-α were also increased in a time-dependent manner at the protein (Fig. 2D–F, *n* = 6) and mRNA level (Additional file 1). It is consistent with the results from NLRP1 inflammasome, suggesting that NLRP1 inflammasome-inflammatory signaling is activated in depressive-like mice. The PI3K/AKT/mTOR signaling pathway is a negative regulator of autophagy, and activated mTOR can suppress the formation of autophagosomes [34]. Interestingly, we found that CSDS exposure induced a gradual increase in the levels of hippocampal p-PI3K, p-AKT and p-mTOR, and they were also activated significantly at 10d after stress stimuli (Fig. 2G–I, *n* = 6). These results indicate that activated NLRP1 inflammasome and PI3K/AKT/mTOR signaling could be contributed to CSDS-induced change in autophagy function in mice.

### NLRP1–PI3K/AKT/mTOR signaling contributes to autophagy dysfunction in depressive-like mice

**Fig. 3:**
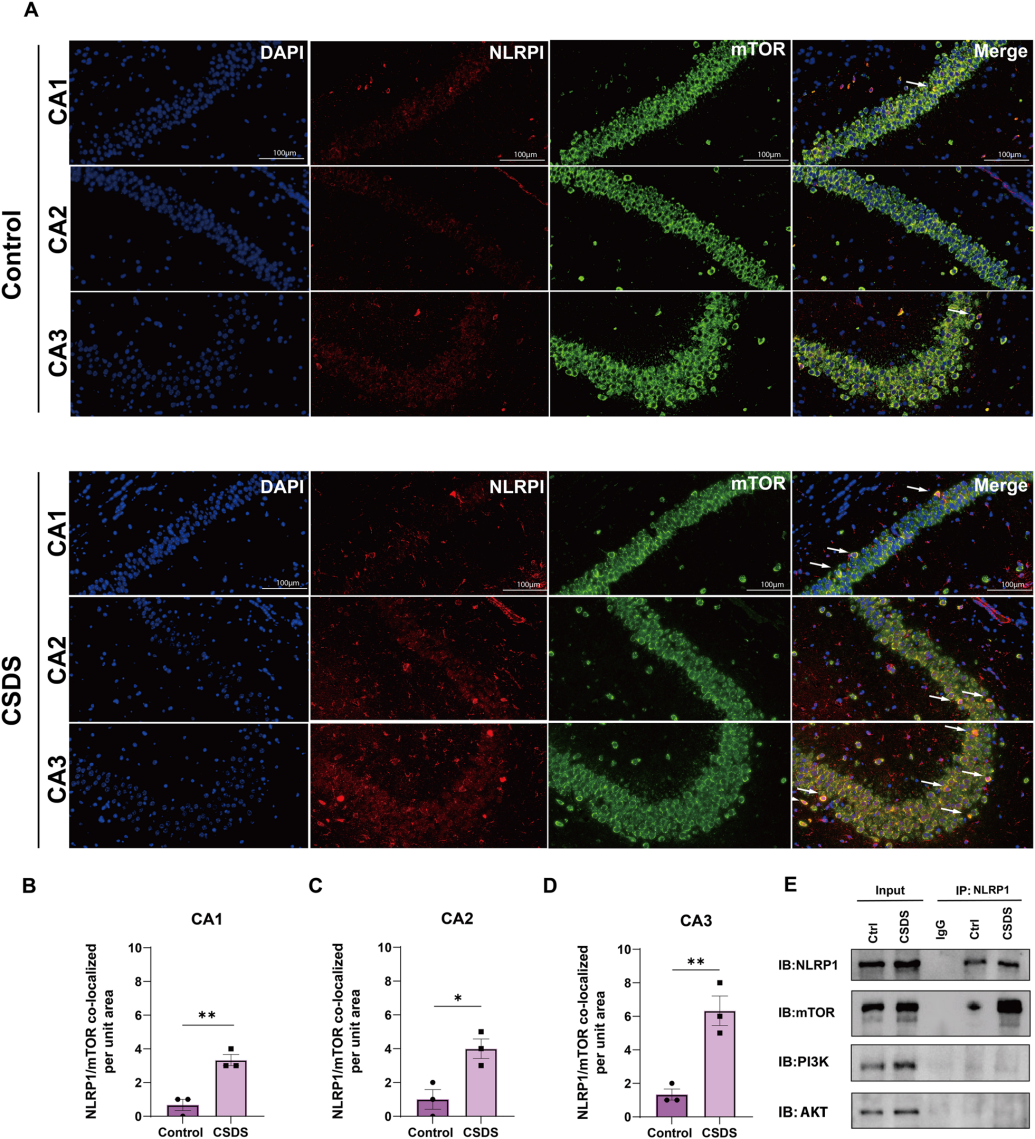
Interaction between NLRP1 and mTOR in the hippocampus. **A**–**D** Representative images (**A**) and quantification of NLRP1/mTOR per unit area show that CSDS (10 d) exposure facilitated the colocalization (arrows) between NLRP1 and mTOR in the hippocampal CA1 (**B**), CA2 (**C**) and CA3 (**D**) region. Scale bar = 100 μm. **E** Representative immunoreactive bands show that NLRP1 is immunoprecipitated with mTOR but not PI3K/AKT and CSDS (10 d) exposure promoted the immunoprecipitation between NLRP1 and mTOR in the hippocampus. IgG is a negative control. Data represented the mean ± SEM, *n* = 3, *p < 0.05, **p < 0.01 vs. control group

**Fig. 4:**
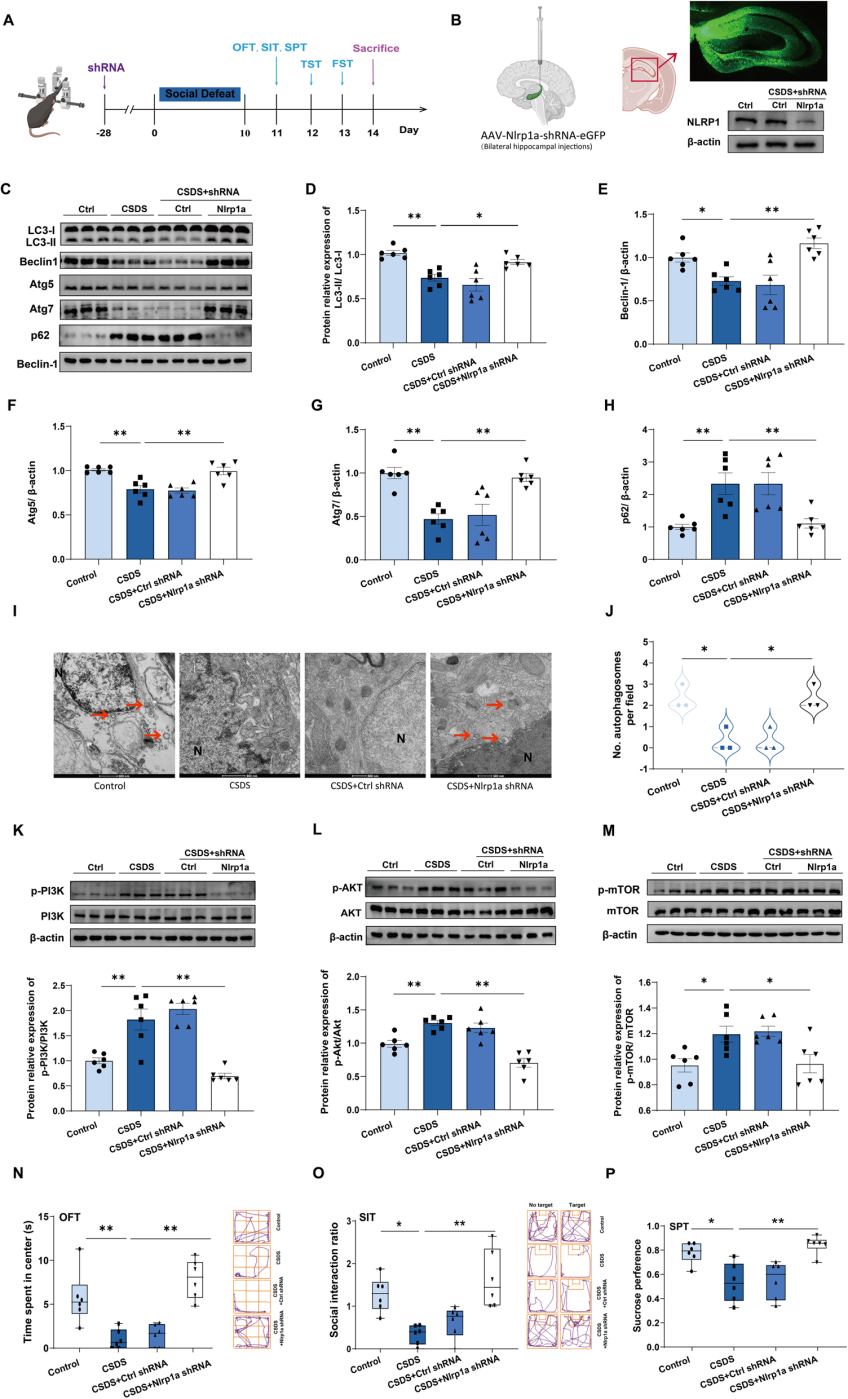
Hippocampal *Nlrp1a* knockdown rescues CSDS-induced autophagy impairment and depressive-like behavior in mice. **A** Scheme of AAV–shRNA infusion, social defeat stress and behavior test. **B** Hippocampal immunofluorescence image and representative immunoreactive bands showing the knockdown efficacy of AAV–Nlrp1a–shRNA. **C**–**H** Representative immunoreactive bands (**C**) and statistical results show that hippocampal *Nlrp1a* knockdown reversed CSDS-induced decrease in the expression of LC3-II/LC3-I (**D**), Beclin-1 (**E**), Atg5 (**F**), Atg7 (**G**) and increase in the expression of p62 (**H**). *n* = 6. **I**, **J** Representative images (**I**) and statistical results (**J**) show that hippocampal *Nlrp1a* knockdown inhibited CSDS-induced decrease in the number of autophagosomes (arrow). *n* = 3. Scale bar = 500 nm. **K**–**M** Representative immunoreactive bands and statistical results show that hippocampal *Nlrp1a* knockdown prevented CSDS-induced increase in the expression of p-PI3K (**K**), p-AKT (**L**) and p-mTOR (**M**). **N**, **O** Representative traces and statistical results show that hippocampal *Nlrp1a* knockdown increased the total moving distance (**N**) and the social interaction (**O**) in depressive-like mice. **P** Statistical results show that hippocampal *Nlrp1a* knockdown increased the sucrose preference in depressive-like mice. *n* = 6. Data were expressed as means ± SEM, *p < 0.05, **p < 0.01 vs. control group or CSDS group

To determine the role of NLRP1 inflammasome in CSDS (10d) induced autophagy dysfunction, we tested the interaction between the NLRP1 inflammasome and mTOR by immunofluorescence and coimmunoprecipitation. As shown in Fig. 3A–D, NLRP1 and mTOR were co-expressed in hippocampal neurons, and CSDS exposure facilitated this colocalization. In addition, our results showed that NLRP1 was notably immunoprecipitated with mTOR. No interactions between NLRP1 and AKT or PI3K were observed (Fig. 3E, *n* = 3). It indicates that NLRP1 inflammasome could be involved in CSDS-induced autophagy impairment via mTOR signaling. To further confirm this, we knocked down *Nlrp1a* successfully to block NLRP1 inflammasome activation by hippocampal AAV–*Nlrp1a*–shRNA injection (Fig. 4A, and B). Our results showed that CSDS-induced autophagy impairment was reversed by hippocampal *Nlrp1a*–shRNA infusion, which was evidenced by the conversion of LC3-I into LC3-II, upregulation of Beclin-1, Atg5, and Atg7 and subsequent degradation of p62 (Fig. 4C–H, *n* = 6). However, control shRNA infusion had no effect on autophagy markers. Similarly, CSDS-induced decrease in the number of autophagosomes was abolished by hippocampal *Nlrp1a* knockdown (Fig. 4I, J, *n* = 3), indicating that NLRP1 inflammasome is responsible for CSDS-induced autophagy impairment. In addition, CSDS-induced increase in the phosphorylation of PI3K, AKT and mTOR was also inhibited by hippocampal *Nlrp1a* knockdown (Fig. 4K–M, *n* = 6), suggesting that NLRP1 inflammasome affects hippocampal autophagy function by PI3K/AKT/mTOR signaling under CSDS. Meanwhile, hippocampal *Nlrp1a* knockdown ameliorated CSDS-induced depressive-like behaviors in mice (Fig. 4N–P, *n* = 6), which is consistent with our previous results [15]. Therefore, our results demonstrate that NLRP1–PI3K/AKT/mTOR signaling contributes to autophagy impairment in depressive-like mice.

### Rapamycin ameliorates CSDS-induced depressive-like behaviors in mice

**Fig. 5:**
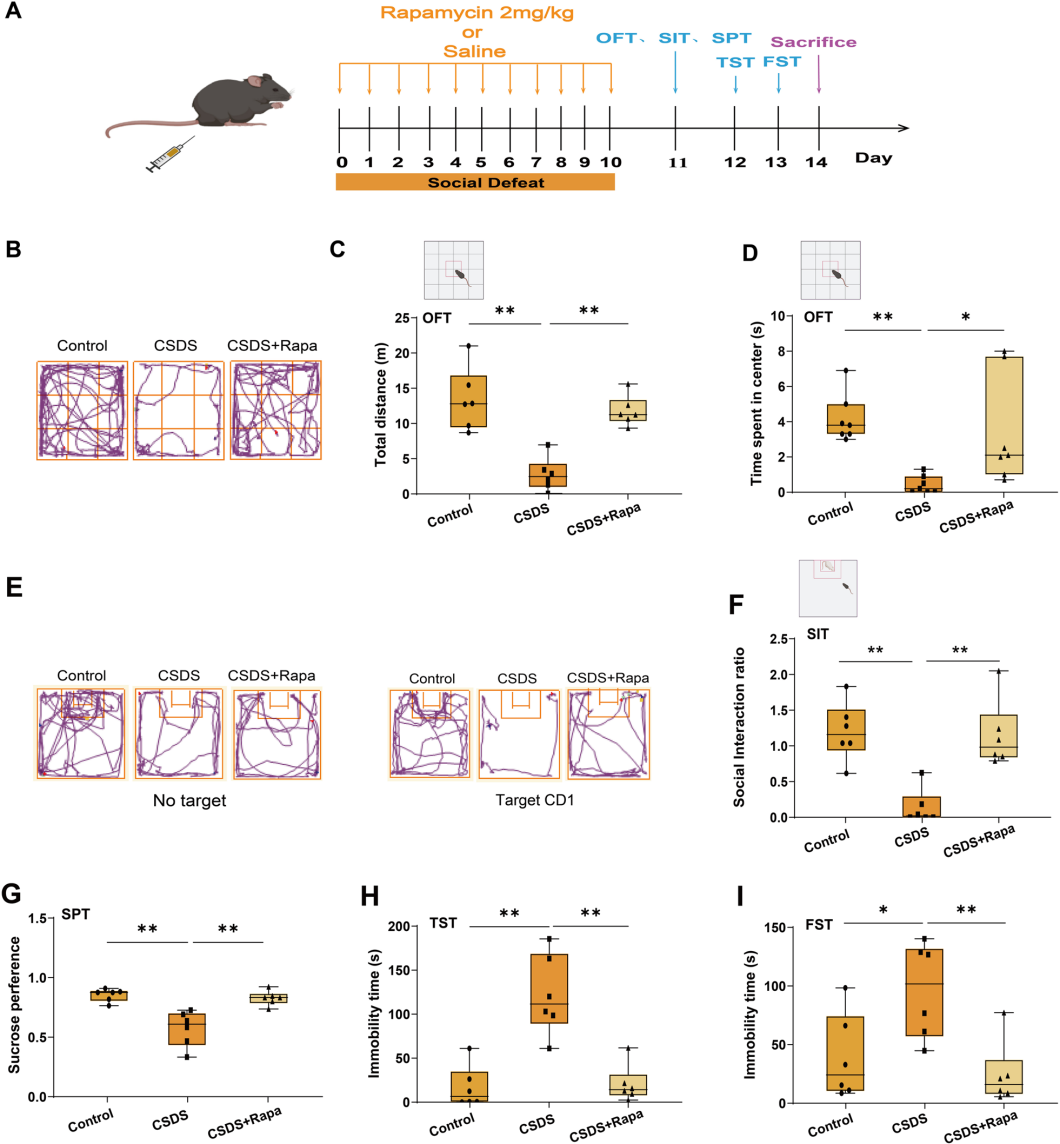
Rapamycin ameliorates CSDS-induced depressive-like behavior in mice. **A** Scheme of rapamycin treatment, social defeat stress and behavior test. **B**–**F** Representative traces and statistical results show that rapamycin increased the total moving distance (**B**–**D**) and the social interaction (**E**, **F**) in depressive-like mice. **G**–**I** Statistical results show that rapamycin increased sucrose preference (**G**) and decreased immobility time during TST (**H**) and FST (**I**) in depressive-like mice. Data are expressed as means ± SEM, *n* = 6. Data were expressed as means ± SEM. *p < 0.05, **p < 0.01 vs. control group or CSDS group

Since NLRP1 inflammasome activation led to autophagy impairment in depressive-like mice, we further aimed to examine the effects of activated autophagy on depressive-like behavior in mice. We used a classical inducer of autophagy rapamycin, which can inhibit the mammalian target of rapamycin (serine/threonine kinase) complex 1 (MTORC1) [35]. The animals were intraperitoneally injected with rapamycin (2 mg/kg body weight per day) during CSDS exposure (10 d) (Fig. 5A). Our results showed that rapamycin treatment abolished CSDS-induced decreased in the locomotor activity, social interaction rate and sucrose preference in mice (Fig. 5B–G, *n* = 6). Meanwhile, rapamycin treatment inhibited the increase in immobility time during the TST and FST (Fig. 5H, I, *n* = 6). These results suggest that rapamycin-induced autophagy alleviates the depressive behavior induced by chronic stress, indicating that autophagy plays a crucial role in the development of depression and could be implicate in NLRP1 inflammasome linked depressive-like behavior.

### Rapamycin inhibits NLRP1 inflammasome activation and exhibits neuroprotective effects in depressive-like mice

**Fig. 6:**
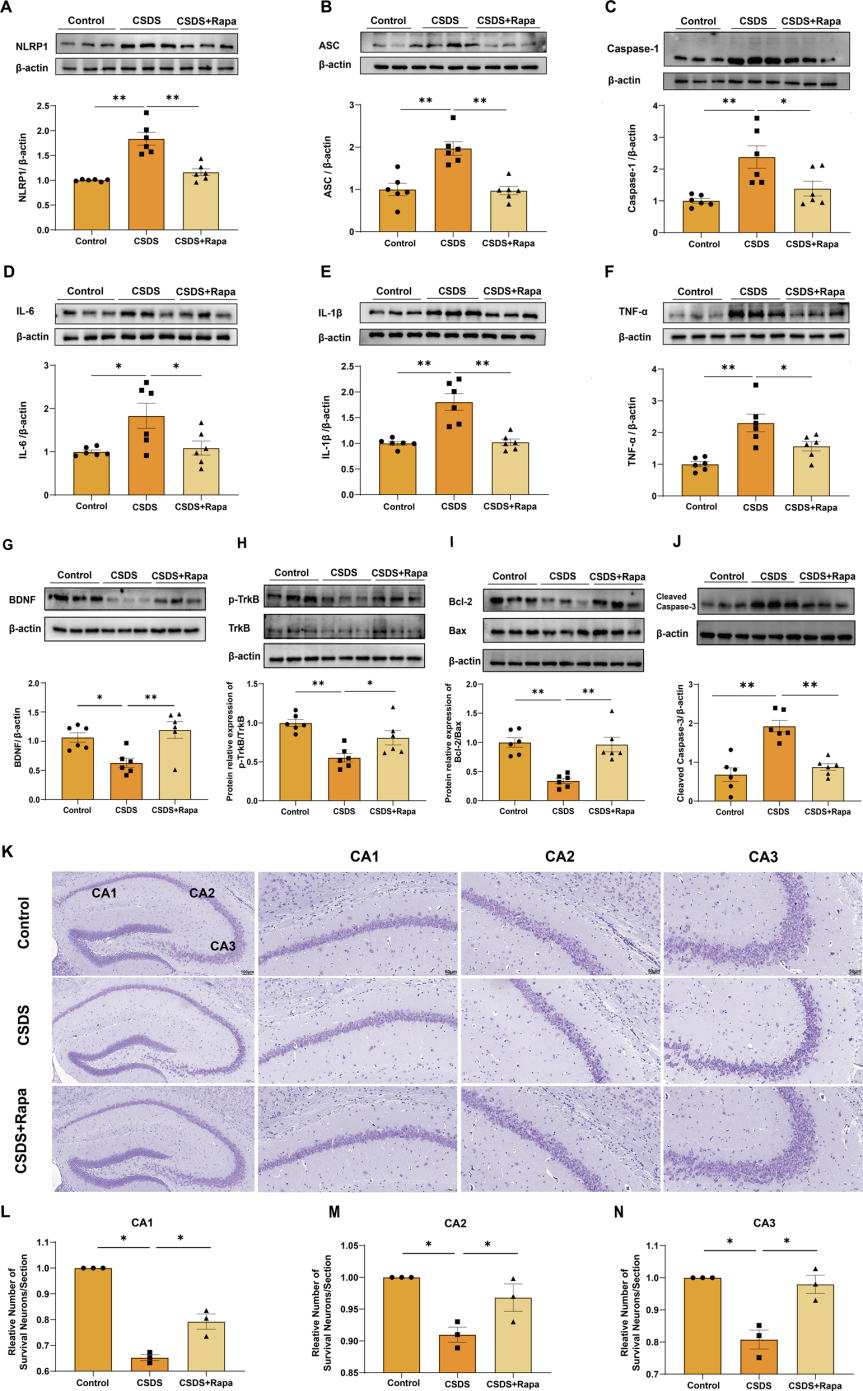
Rapamycin inhibits NLRP1 inflammasome signaling and exhibits a neuroprotective effect in depressive-like mice. **A–F** Representative immunoreactive bands and statistical results show that rapamycin inhibited CSDS-induced increase in the expression of hippocampal NLRP1 (**A**), ASC (**B**), Caspase1 (**C**), IL-6 (**D**), IL-1β (**E**), TNF-α (**F**). *n* = 6. **G**–**I** Representative immunoreactive bands and statistical results show that rapamycin restored the levels of hippocampal BDNF (**G**), p-TrkB (**H**), Bcl-2/Bax (**I**) and cleaved caspase-3 (**J**) in depressive-like mice. *n* = 6. **K**–**N** Representative images (**K**) and statistical results (**L**–**N**) show that rapamycin prevented CSDS-induced decrease in the number of NeuN in hippocampal CA1, CA2, CA3 region. *n* = 3, scale bar = 50 μm. Data expressed as mean ± SEM, *p < 0.05, **p < 0.01 vs. control group or CSDS group

To further determine the role of autophagy in NLRP1 inflammasome-mediated depressive-like behavior in mice, we tested the effects of autophagy activation on NLRP1 inflammasome. We found that rapamycin treatment significantly decreased the protein expression of NLRP1, ASC, and caspase-1 (Fig. 6A–C, *n* = 6). In addition, rapamycin treatment markedly inhibited CSDS-induced (10d) increase in the expression of IL-6, IL-1β, and TNF-α at the protein (Fig. 6D–F, *n* = 6) and mRNA level (Additional file 2). These results suggest that activated autophagy can inhibit the activity of NLRP1 inflammasome and inflammatory response in depressive-like mice. BDNF–TrkB signaling plays a vital role in depression, where BDNF and p-TrkB levels are significantly low [36, 37]. Previous studies have reported that BDNF mediates the regulation of apoptosis by regulating members of the Bcl-2 family [38]. Our results showed that hippocampal BDNF and p-TrkB expression was significantly decreased at 10d after CSDS exposure (Fig. 6G, H, *n* = 6), while rapamycin treatment inhibited these effects. Similarly, the CSDS-induced decrease in the Bcl-2/Bax ratio and increase in the expression of cleaved caspase-3 were inhibited by rapamycin treatment (Fig. 6I, J, *n* = 6). In addition, CSDS exposure caused a significant decrease in the number of NeuN-positive neurons in the hippocampus. This effect was also abolished by rapamycin treatment (Fig. 6K–N, *n* = 3). This indicates that rapamycin treatment may mitigate the stress-induced neuronal alterations in these specific hippocampal subfields. Taken together, our results suggest that activated autophagy blocks NLRP1 inflammasome-driven inflammatory process, alleviates depressive-like behavior and exerts a neuroprotective effect, indicating that autophagy dysfunction is involved in NLRP1 inflammasome-linked depressive-like behavior induced by CSDS in mice.

## Discussion

NLRP1 inflammasome and autophagy have been reported to implicate in the development of depression [39]. However, the mechanistic interplay between NLRP1 inflammasome, autophagy, and depression is still largely unknown. To investigate this, we studied the effect of CSDS exposure on autophagy and its role in NLRP1 inflammasome-linked depressive-like behavior in mice. Our results showed that CSDS exposure led to a bidirectional change in autophagy function, activated in the initial period (3d) and impaired in the later stage (10d). Hippocampal *Nlrp1a* knockdown rescued defective autophagy by inhibiting PI3K/AKT/mTOR signaling. Moreover, autophagy inducer rapamycin abolished NLRP1inflammasome-mediated inflammatory response, ameliorated depressive-like behavior and exerted a neuroprotective effect. These results suggest that autophagy dysfunction could be involved in NLRP1 inflammasome-linked depressive-like behavior in mice.

Autophagy is an intracellular catabolic pathway that degrades certain amounts of waste proteins, toxic aggregates, and damaged organelles, which is essential for cellular homeostasis [16]. Apart from a regulated cellular degradation pathway, autophagy is also represented as a stress response pathway [40]. Accumulating evidence indicates that normal autophagy could be associated with physiological stress, and excessive or prolonged stress would induce defective autophagy [41, 42]. Studies have suggested that a reduction in autophagy may affect multiple cell functions and ultimately result in MDD, and antidepressant treatment exerted an enhanced autophagic pathway [43, 44]. In contrast, some studies exhibited that neuroinflammation triggered autophagy activation resulting in depressive symptoms in animal models [45]. Thus, the definitive role of autophagy in depression and its underlying molecular mechanisms remain to be elucidated. Here, we tested the changes of hippocampal autophagy markers in mice with different durations of CSDS. We found that CSDS exposure induced a dynamic change in autophagy function. It was evoked in the initial period of CSDS and impaired at the later stage. Interestingly, the depressive-like behavior was not observed in the initial period of CSDS but was induced at the later stage, which was in consistent with the dynamic change of autophagy. In addition, CSDS-induced decrease in the expression of LC3 II/LC3-I was reversed by autophagy inducer rapamycin (See Additional file 3), suggesting autophagic flux could be decreased in depressive-like mice. However, further study will be made to comprehensive assess the change of autophagic flux during depression in future**.** Taken together, our results indicate that normal autophagy is beneficial in antidepressants and dysfunctional autophagy could lead to depressive-like behavior.

Inflammation is usually a protective response of the body to harmful stimuli, including infections, physical injury, or other insults [46]. An acute inflammatory response in the short term is usually beneficial, while a more enormous or prolonged inflammatory response may lead to negative consequences [47]. As a key component of the innate immune response, inflammasomes exhibited a critical role in controlling the production and the release of proinflammatory cytokines. The activity of inflammasomes and autophagy are considered as two fundamental cellular responses to a variety of stresses [48]. The mutual relationship between inflammasomes and autophagy has been reported in many studies. Inflammasomes can activate autophagy as well as lead to defective autophagy. Meanwhile, autophagy can prevent inflammasome activation and the subsequent release of proinflammatory cytokines. Depletion of autophagy by genetic or pharmacological means enhanced inflammatory response and the release of proinflammatory cytokines [43, 49–51]. Increasing evidence has shown an interplay of inflammasome and autophagy implicated in the pathological process of many neurological disorders, such as Alzheimer’s disease (AD), Parkinson’s disease (PD) and multiple sclerosis (MS) [52]. Recent studies showed that NLRP3 inflammasome interacts with autophagy in depression [53]. However, the interplay of NLRP1 inflammasome and autophagy in depression remains unclear. To determine this, we investigated their interaction in the CSDS model by genetic and pharmacological methods. Unlike the bidirectional change in autophagy function, CSDS exposure induced a gradual increase in the expression of NLRP1 inflammasome complexes and proinflammatory cytokines. It indicates that short-term stress stimuli could trigger autophagy to inhibit the activity of NLRP1 inflammasome and inflammatory pathway, while prolonged stress stimuli caused excessive inflammatory response resulting in autophagy impairment. Interestingly, hippocampal *Nlrp1a* knockdown abolished CSDS-induced autophagy impairment in depressive-like mice. Moreover, rapamycin-induced autophagy inhibited NLRP1 inflammasome-driven inflammatory response and ameliorated depressive-like behavior in mice. These results demonstrate that defective autophagy could contribute to NLRP1 inflammasome-linked depressive-like behavior.

mTOR, a serine/threonine kinase, plays a vital role in the regulation of autophagy. It can be activated by upstream PI3K/AKT signaling and cause a reduction in autophagy [54]. To further study the underlying molecular mechanism of autophagy dysfunction in depression, we examined the interaction between NLRP1inflammasome and PI3K/AKT/mTOR signaling pathway. Our results showed that NLRP1 and mTOR colocalized and coimmunoprecipitated in the hippocampal neurons of mice. Hippocampal *Nlrp1a* knockdown blocked the activity of mTOR induced by CSDS, indicating that mTOR signaling could be implicated in the autophagy regulation of NLRP1 inflammasome. Meanwhile, we found that NLRP1 was not immunoprecipitated with PI3K or AKT. However, CSDS-induced activity of PI3K/AKT signaling was also inhibited by hippocampal *Nlrp1a* knockdown. Previous studies have shown that PI3K/AKT signaling can be activated by IL-1β, which is recognized as the main mediators among numerous pro-inflammatory cytokines produced by inflammasome activation [55, 56]. Thus, NLRP1 inflammasome could regulate mTOR by IL-1β linked PI3K/AKT signaling besides their direct interaction. These results suggest that NLRP1 inflammasome could regulate autophagy function by PI3K/AKT/mTOR signaling pathway in depression.

Memory and cognitive impairment are the core features of depression, which are associated with BDNF level and neuronal apoptosis [57, 58]. Hippocampus is the major structure involved in learning, memory and affective behavior. Accumulating evidence showed that brain BDNF level is reduced in experimental animals and patients with depression [36, 37]. Our previous study also showed that chronic stress significantly reduced the expression of hippocampal BDNF, while hippocampal *Nlrp1a* knockdown reversed BDNF levels. Here, we found that rapamycin-induced autophagy significantly mitigated CSDS-induced inflammatory responses, restored hippocampal BDNF and p-TrkB levels and ameliorate CSDS-induced depressive-like behavior in mice. The antiapoptotic factor Bcl-2 has been identified as an interaction partner of Beclin-1, allowing Bcl-2 to inhibit both apoptosis and autophagy [59]. Our results also showed that rapamycin treatment significantly reversed the decreased ratio of hippocampal Bcl2/Bax and the increased expression of cleaved caspase-3 in CSDS-exposed mice. In addition, autophagy activation reduced the neuronal apoptosis induced by CSDS. These results suggest that the activity of autophagy can restore BDNF level, reduce neuronal apoptosis and exert a neuroprotective effect in depressive-like mice.

Although our findings revealed the interaction between NLRP1 inflammasome and autophagy in CSDS-induced depression model, the underlying molecular mechanisms was not fully elucidated. Recent study showed that NLRs interacts with autophagy proteins, and the NACHT structural domain interacts with Beclin-1, a protein involved in autophagy initiation [60]. However, whether NLRP1 interact with autophagy-related protein directly is still unclear. In addition, it should be noted that aging is an important risk element for many nervous system diseases, including depression, and aging also has an influence on autophagy processes [61]. Our previous study showed that aging promoted chronic stress-induced depressive-like behavior by activating NLRP1 inflammasome driven inflammatory signaling in mice [62]. Therefore, aging could be a key factor in the interplay of NLRP1 inflammasome and autophagy, and the precise mechanism under their interaction in depression deserves further investigation in the future.

## Conclusions

Our results demonstrated that CSDS exposure caused a dynamic change in autophagy function. NLRP1–PI3K/AKT/mTOR signaling is involved in CSDS-induced defective autophagy. In addition, activated autophagy blocked NLRP1 inflammasome signaling and ameliorated depressive-like behavior in mice. Taken together, our findings suggested that autophagy dysfunction contributes to NLRP1 inflammasome-linked depressive-like behavior in mice and the regulation of autophagy could be a valuable therapeutic strategy to treat chronic stress-induced depressive-like behavior. However, further studies will be made to clarify the precise mechanism of the interplay of NLRP1 inflammasome and autophagy in depression.

### Supplementary Information

**Additional file 1. Figure S1.** Effects of different time courses of CSDS exposure on the mRNA levels of proinflammatory cytokines in mice. Statistical results show that CSDS exposure increased the mRNA levels of hippocampal IL-6 (**A**), IL-1β (**B**) and TNF-α (**C**) in a time dependent manner. Data represented the mean ± SEM. n = 6, * p < 0.05 *vs* control group.

**Additional file 2: Figure S2.** Rapamycin decreases the mRNA levels of proinflammatory cytokines in depressive-like mice. Statistical results show that rapamycin inhibited CSDS-induced increase in the mRNA levels of hippocampal IL-6 (**A**), IL-1β (**B**) and TNF-α (**C**). Data represented the mean ± SEM. n = 6, *p < 0.05, **p < 0.01 *vs* control group or CSDS group.

**Additional file 3: Figure S3.** Rapamycin increases the protein expression of LC3-II/LC3-I in depressive-like mice. Representative immunoreactive bands and statistical results show that rapamycin inhibited CSDS-induced decrease in the expression of hippocampal LC3-II/LC3-I. Data were expressed as mean ± SEM. n = 6, *p < 0.05, **p < 0.01 *vs* control group or CSDS group.

## Data Availability

The data sets used and/or analyzed during the current study are available from the corresponding author on reasonable request.

## Abbreviations

AAV: Adeno-associated virus
AD: Alzheimer’s disease
ASC: Apoptosis-associated speck-like protein containing a caspase-activating recruitment domain
CNS: Central nervous system
Co-IP: Coimmunoprecipitation
FST: Forced swim test
MDD: Major depressive disorder
MS: Multiple sclerosis
NLRP1: Nucleotide-binding oligomerization domain, leucine-rich repeat and pyrin domain-containing 1
OFT: Open field test
PD: Parkinson’s disease
SIT: Social interaction test
SPT: Sucrose preference test
TEM: Transmission electron microscopy
TST: Tail-suspension test

## References

[1] Belmaker RH, Agam G (2008). Major depressive disorder. N Engl J Med.

[2] Walker ER, McGee RE, Druss BG (2015). Mortality in mental disorders and global disease burden implications: a systematic review and meta-analysis. JAMA Psychiat.

[3] Smith K (2014). Mental health: a world of depression. Nature.

[4] Zeier Z, Carpenter LL, Kalin NH, Rodriguez CI, McDonald WM, Widge AS, Nemeroff CB (2018). Clinical implementation of pharmacogenetic decision support tools for antidepressant drug prescribing. Am J Psychiatry.

[5] Raison CL, Capuron L, Miller AH (2006). Cytokines sing the blues: inflammation and the pathogenesis of depression. Trends Immunol.

[6] Miller AH, Maletic V, Raison CL (2009). Inflammation and its discontents: the role of cytokines in the pathophysiology of major depression. Biol Psychiatry.

[7] Kappelmann N, Lewis G, Dantzer R, Jones PB, Khandaker GM (2018). Antidepressant activity of anti-cytokine treatment: a systematic review and meta-analysis of clinical trials of chronic inflammatory conditions. Mol Psychiatry.

[8] Kohler-Forsberg O, Lydholm CN, Hjorthoj C, Nordentoft M, Mors O, Benros ME (2019). Efficacy of anti-inflammatory treatment on major depressive disorder or depressive symptoms: meta-analysis of clinical trials. Acta Psychiatr Scand.

[9] Adzic M, Brkic Z, Mitic M, Francija E, Jovicic MJ, Radulovic J, Maric NP (2018). Therapeutic strategies for treatment of inflammation-related depression. Curr Neuropharmacol.

[10] Liu CH, Zhang GZ, Li B, Li M, Woelfer M, Walter M, Wang L (2019). Role of inflammation in depression relapse. J Neuroinflammation.

[11] Schroder K, Tschopp J (2010). The inflammasomes. Cell.

[12] Strowig T, Henao-Mejia J, Elinav E, Flavell R (2012). Inflammasomes in health and disease. Nature.

[13] Davis BK, Wen H, Ting JP (2011). The inflammasome NLRs in immunity, inflammation, and associated diseases. Annu Rev Immunol.

[14] Gao B, Wu Y, Yang YJ, Li WZ, Dong K, Zhou J, Yin YY, Huang DK, Wu WN (2018). Sinomenine exerts anticonvulsant profile and neuroprotective activity in pentylenetetrazole kindled rats: involvement of inhibition of NLRP1 inflammasome. J Neuroinflammation.

[15] Song AQ, Gao B, Fan JJ, Zhu YJ, Zhou J, Wang YL, Xu LZ, Wu WN (2020). NLRP1 inflammasome contributes to chronic stress-induced depressive-like behaviors in mice. J Neuroinflammation.

[16] Mizushima N, Levine B (2020). Autophagy in human diseases. N Engl J Med.

[17] Menzies FM, Fleming A, Caricasole A, Bento CF, Andrews SP, Ashkenazi A, Fullgrabe J, Jackson A, Jimenez Sanchez M, Karabiyik C (2017). Autophagy and neurodegeneration: pathogenic mechanisms and therapeutic opportunities. Neuron.

[18] Sarkar C, Zhao Z, Aungst S, Sabirzhanov B, Faden AI, Lipinski MM (2014). Impaired autophagy flux is associated with neuronal cell death after traumatic brain injury. Autophagy.

[19] Liu T, Han S, Dai Q, Zheng J, Liu C, Li S, Li J (2019). IL-17A-mediated excessive autophagy aggravated neuronal ischemic injuries via Src-PP2B-mTOR pathway. Front Immunol.

[20] Li G, Sherchan P, Tang Z, Tang J (2021). Autophagy & phagocytosis in neurological disorders and their possible cross-talk. Curr Neuropharmacol.

[21] Jia J, Le W (2015). Molecular network of neuronal autophagy in the pathophysiology and treatment of depression. Neurosci Bull.

[22] Saitoh T, Fujita N, Jang MH, Uematsu S, Yang B-G, Satoh T, Omori H, Noda T, Yamamoto N, Komatsu M, et al. Loss of the autophagy protein Atg16L1 enhances endotoxin-induced IL-1β production. 10.1038/nature07383 18849965

[23] Matsuzawa-Ishimoto Y, Hwang S, Cadwell K (2018). Autophagy and Inflammation. Annu Rev Immunol.

[24] Levine B, Mizushima N, Virgin HW (2011). Autophagy in immunity and inflammation. Nature.

[25] Cadwell K (2016). Crosstalk between autophagy and inflammatory signalling pathways: balancing defence and homeostasis. Nat Rev Immunol.

[26] Li M, Sun T, Wu X, An P, Wu X, Dang H (2021). Autophagy in the HTR-8/SVneo cell oxidative stress model is associated with the NLRP1 inflammasome. Oxid Med Cell Longev.

[27] Yang Y, Li J, Rao T, Fang Z, Zhang J (2021). The role and mechanism of hyperoside against myocardial infarction in mice by regulating autophagy via NLRP1 inflammation pathway. J Ethnopharmacol.

[28] Nie X, Kitaoka S, Tanaka K, Segi-Nishida E, Imoto Y, Ogawa A, Nakano F, Tomohiro A, Nakayama K, Taniguchi M (2018). The innate immune receptors TLR2/4 mediate repeated social defeat stress-induced social avoidance through prefrontal microglial activation. Neuron.

[29] Golden SA, Covington HE, Berton O, Russo SJ (2011). A standardized protocol for repeated social defeat stress in mice. Nat Protocol.

[30] Jiang N, Zhang BY, Dong LM, Lv JW, Lu C, Wang Q, Fan LX, Zhang HX, Pan RL, Liu XM (2018). Antidepressant effects of dammarane sapogenins in chronic unpredictable mild stress-induced depressive mice. Phytother Res.

[31] Kroemer G, Marino G, Levine B (2010). Autophagy and the integrated stress response. Mol Cell.

[32] Pierone BC, Pereira CA, Garcez ML, Kaster MP (2020). Stress and signaling pathways regulating autophagy: from behavioral models to psychiatric disorders. Exp Neurol.

[33] Ali T, Rahman SU, Hao Q, Li W, Liu Z, Ali Shah F, Murtaza I, Zhang Z, Yang X, Liu G, Li S (2020). Melatonin prevents neuroinflammation and relieves depression by attenuating autophagy impairment through FOXO3a regulation. J Pineal Res.

[34] Heras-Sandoval D, Perez-Rojas JM, Hernandez-Damian J, Pedraza-Chaverri J (2014). The role of PI3K/AKT/mTOR pathway in the modulation of autophagy and the clearance of protein aggregates in neurodegeneration. Cell Signal.

[35] Chen K, Zheng Y, Wei JA, Ouyang H, Huang X, Zhang F, Lai CSW, Ren C, So KF, Zhang L (2019). Exercise training improves motor skill learning via selective activation of mTOR. Sci Adv.

[36] Tripp A, Oh H, Guilloux JP, Martinowich K, Lewis DA, Sibille E (2012). Brain-derived neurotrophic factor signaling and subgenual anterior cingulate cortex dysfunction in major depressive disorder. Am J Psychiatry.

[37] Martinowich K, Manji H, Lu B (2007). New insights into BDNF function in depression and anxiety. Nat Neurosci.

[38] Zhou CH, Zhang YH, Xue F, Xue SS, Chen YC, Gu T, Peng ZW, Wang HN (2017). Isoflurane exposure regulates the cell viability and BDNF expression of astrocytes via upregulation of TREK1. Mol Med Rep.

[39] Gassen NC, Rein T (2019). Is there a role of autophagy in depression and antidepressant action?. Front Psychiatry.

[40] Kumsta C, Chang JT, Schmalz J, Hansen M (2017). Hormetic heat stress and HSF-1 induce autophagy to improve survival and proteostasis in *C. elegans*. Nat Commun.

[41] Puri D, Subramanyam D (2019). Stress—(self) eating: epigenetic regulation of autophagy in response to psychological stress. FEBS J.

[42] Gu HF, Nie YX, Tong QZ, Tang YL, Zeng Y, Jing KQ, Zheng XL, Liao DF (2014). Epigallocatechin-3-gallate attenuates impairment of learning and memory in chronic unpredictable mild stress-treated rats by restoring hippocampal autophagic flux. PLoS ONE.

[43] Alcocer-Gomez E, Casas-Barquero N, Williams MR, Romero-Guillena SL, Canadas-Lozano D, Bullon P, Sanchez-Alcazar JA, Navarro-Pando JM, Cordero MD (2017). Antidepressants induce autophagy dependent-NLRP3-inflammasome inhibition in Major depressive disorder. Pharmacol Res.

[44] Gulbins A, Schumacher F, Becker KA, Wilker B, Soddemann M, Boldrin F, Muller CP, Edwards MJ, Goodman M, Caldwell CC (2018). Antidepressants act by inducing autophagy controlled by sphingomyelin-ceramide. Mol Psychiatry.

[45] Jiang G, Wang Y, Liu Q, Gu T, Liu S, Yin A, Zhang L (2022). Autophagy:a new mechanism for esketamine as a depression therapeutic. Neuroscience.

[46] Kohler O, Krogh J, Mors O, Benros ME (2016). Inflammation in depression and the potential for anti-inflammatory treatment. Curr Neuropharmacol.

[47] Skaper SD, Facci L, Zusso M, Giusti P (2018). An inflammation-centric view of neurological disease: beyond the neuron. Front Cell Neurosci.

[48] Yuk JM, Jo EK (2013). Crosstalk between autophagy and inflammasomes. Mol Cells.

[49] Cosin-Roger J, Simmen S, Melhem H, Atrott K, Frey-Wagner I, Hausmann M, de Valliere C, Spalinger MR, Spielmann P, Wenger RH (2017). Hypoxia ameliorates intestinal inflammation through NLRP3/mTOR downregulation and autophagy activation. Nat Commun.

[50] Li JR, Xu HZ, Nie S, Peng YC, Fan LF, Wang ZJ, Wu C, Yan F, Chen JY, Gu C (2017). Fluoxetine-enhanced autophagy ameliorates early brain injury via inhibition of NLRP3 inflammasome activation following subrachnoid hemorrhage in rats. J Neuroinflammation.

[51] Shi CS, Shenderov K, Huang NN, Kabat J, Abu-Asab M, Fitzgerald KA, Sher A, Kehrl JH (2012). Activation of autophagy by inflammatory signals limits IL-1beta production by targeting ubiquitinated inflammasomes for destruction. Nat Immunol.

[52] Panda C, Mahapatra RK (2023). Bi-directional relationship between autophagy and inflammasomes in neurodegenerative disorders. Cell Mol Neurobiol.

[53] Geng J, Liu J, Yuan X, Liu W, Guo W (2019). Andrographolide triggers autophagy-mediated inflammation inhibition and attenuates chronic unpredictable mild stress (CUMS)-induced depressive-like behavior in mice. Toxicol Appl Pharmacol.

[54] Querfurth H, Lee HK (2021). Mammalian/mechanistic target of rapamycin (mTOR) complexes in neurodegeneration. Mol Neurodegener.

[55] Xu K, He Y, Moqbel SAA, Zhou X, Wu L, Bao J (2021). SIRT3 ameliorates osteoarthritis via regulating chondrocyte autophagy and apoptosis through the PI3K/Akt/mTOR pathway. Int J Biol Macromol.

[56] Norman GJ, Karelina K, Zhang N, Walton JC, Morris JS, Devries AC (2010). Stress and IL-1beta contribute to the development of depressive-like behavior following peripheral nerve injury. Mol Psychiatry.

[57] Rock PL, Roiser JP, Riedel WJ, Blackwell AD (2014). Cognitive impairment in depression: a systematic review and meta-analysis. Psychol Med.

[58] Freeman LC, Ting JP (2016). The pathogenic role of the inflammasome in neurodegenerative diseases. J Neurochem.

[59] Fernandez AF, Sebti S, Wei Y, Zou Z, Shi M, McMillan KL, He C, Ting T, Liu Y, Chiang WC (2018). Disruption of the beclin 1-BCL2 autophagy regulatory complex promotes longevity in mice. Nature.

[60] Biasizzo M, Kopitar-Jerala N (2020). Interplay between NLRP3 inflammasome and autophagy. Front Immunol.

[61] Aman Y, Schmauck-Medina T, Hansen M, Morimoto RI, Simon AK, Bjedov I, Palikaras K, Simonsen A, Johansen T, Tavernarakis N (2021). Autophagy in healthy aging and disease. Nat Aging.

[62] Zhu Y-J, Fan J-J, Wu F-Y, Zhang M, Song A-Q, Li Y, Li Y-K, Wu W-N (2022). Aging promotes chronic stress-induced depressive-like behavior by activating NLRP1 inflammasome-driven inflammatory signaling in mice. Inflammation.

