# Supplementary material for: Autophagy dysfunction contributes to NLRP1 inflammasome-linked depressive-like behaviors in mice
Source: J Neuroinflammation. 2024 Jan 4;21:6. doi: 10.1186/s12974-023-02995-4 (PMC10765763; doi:10.1186/s12974-023-02995-4)
Supplement: Supplementary file 1 — Additional file 1. Figure S1. Effects of different time courses of CSDS exposure on the mRNA levels of proinflammatory cytokines in mice. Statistical results show that CSDS exposure increased the mRNA levels of hippocampal IL-6 (A), IL-1β (B) and TNF-α (C) in a time dependent manner. Data represented the mean ± SEM. n = 6, * p < 0.05 vs control group. [file 12974_2023_2995_MOESM1_ESM.pdf]

Figure S1

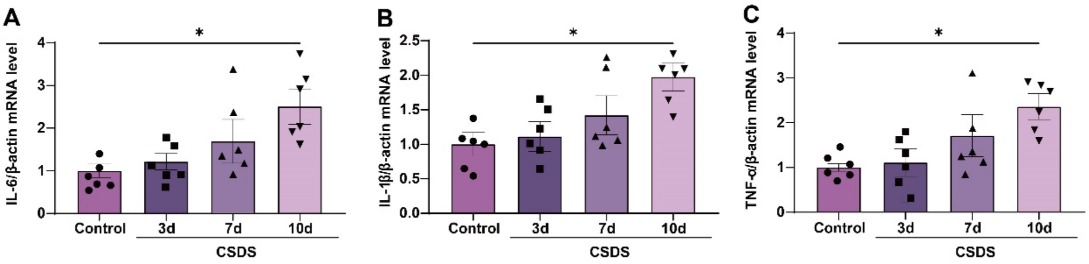

Fig. S1 Effects of different time courses of CSDS on the mRNA levels of proinflammatory cytokines in mice. Statistical results show that CSDS increased the mRNA levels of hippocampal IL-6 (A), IL-1β(B) and TNF-α (C) in a time-dependent manner. Data represented the mean ± SEM. n = 6, $*p < 0.05$ *vs* control group.
