# Supplementary material for: Autophagy dysfunction contributes to NLRP1 inflammasome-linked depressive-like behaviors in mice
Source: J Neuroinflammation. 2024 Jan 4;21:6. doi: 10.1186/s12974-023-02995-4 (PMC10765763; doi:10.1186/s12974-023-02995-4)
Supplement: Supplementary file 2 — Additional file 2: Figure S2. Rapamycin decreases the mRNA levels of proinflammatory cytokines in depressive-like mice. Statistical results show that rapamycin inhibited CSDS-induced increase in the mRNA levels of hippocampal IL-6 (A), IL-1β (B) and TNF-α (C). Data represented the mean ± SEM. n = 6, *p < 0.05, **p < 0.01 vs control group or CSDS group. [file 12974_2023_2995_MOESM2_ESM.pdf]

Figure S2

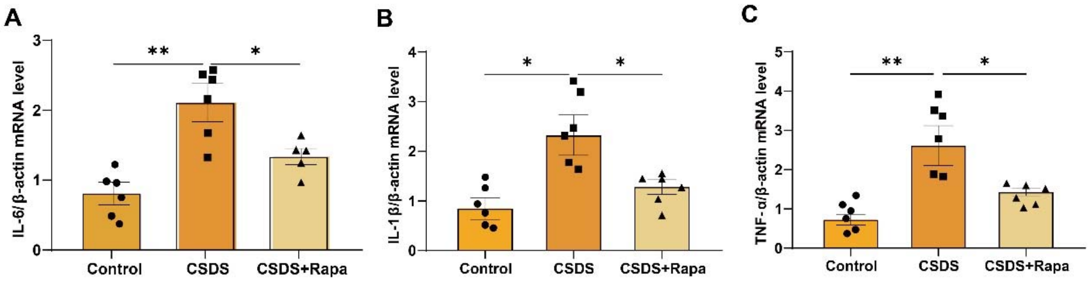

Fig. S2 Rapamycin decreases the mRNA levels of proinflammatory cytokines in depressive-like mice. Statistical results show that rapamycin inhibited CSDS-induced increase in the mRNA levels of hippocampal IL-6 (A), IL-1β(B) and TNF-α (C). Data represented the mean ± SEM. n = 6, *$p < 0.05$, **$p < 0.01$ *vs* control group or CSDS group.
