# Supplementary material for: Autophagy dysfunction contributes to NLRP1 inflammasome-linked depressive-like behaviors in mice
Source: J Neuroinflammation. 2024 Jan 4;21:6. doi: 10.1186/s12974-023-02995-4 (PMC10765763; doi:10.1186/s12974-023-02995-4)
Supplement: Supplementary file 3 — Additional file 3: Figure S3. Rapamycin increases the protein expression of LC3-II/LC3-I in depressive-like mice. Representative immunoreactive bands and statistical results show that rapamycin inhibited CSDS-induced decrease in the expression of hippocampal LC3-II/LC3-I. Data were expressed as mean ± SEM. n = 6, *p < 0.05, **p < 0.01 vs control group or CSDS group. [file 12974_2023_2995_MOESM3_ESM.pdf]

Figure S3

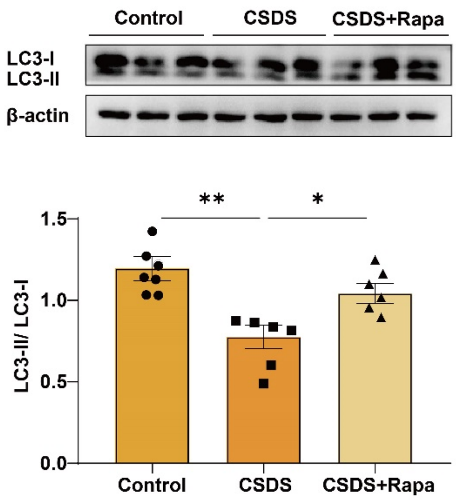

Fig. S3 Rapamycin increases the protein expression of LC3 in depressive-like mice. Representative immunoreactive bands and statistical results show that rapamycin inhibited CSDS-induced decrease in the ratio of hippocampal LC3II/LC3I. Data were expressed as mean ± SEM. n = 6, *$p < 0.05$, **$p < 0.01$ *vs* control group or CSDS group.
